# Magnitude and trend of HIV and *Treponema pallidum* infections among blood donors in Offinso-North District, Ghana: a nine-year retrospective, cross-sectional study

**DOI:** 10.4314/ahs.v22i1.55

**Published:** 2022-03

**Authors:** Charles Nkansah, Dorcas Serwaa, Felix Osei-Boakye, Richard Owusu-Ampomah

**Affiliations:** 1 Department of Medical Diagnostics, Faculty of Allied Health Sciences, Kwame Nkrumah University of Science and Technology, Kumasi, Ghana; Clinical Laboratory Department, Nkenkaasu District Hospital, Nkenkaasu, Ghana; 2 Reproductive Biology Unit, Department of Obstetrics and Gynaecology, College of Medicine, Pan African University of Life and Earth Sciences Institute (PAULESI), University of Ibadan, Ibadan, Nigeria; 3 Department of Medical Diagnostics, Faculty of Allied Health Sciences, Kwame Nkrumah University of Science and Technology, Kumasi, Ghana; Clinical Laboratory Department, Mankranso District Hospital, Mankranso, Ghana; 4 Clinical Laboratory Department, Asokwa Children's Hospital, Kumasi, Ghana

**Keywords:** Blood donors, HIV, magnitude, trend, *Treponema pallidum*

## Abstract

**Introduction:**

Blood transfusion poses a high public health risk to recipients; hence no effort recommended to eradicate or minimize the danger of transmitting the infections.

Reproductive Biology should be underestimated at minimizing the risk of TTIs. This study determined the prevalence and trend of HIV and syphilis infections in voluntary blood donors.

**Method:**

A retrospective analysis of secondary data from consecutive prospective voluntary blood donors who accessed Nkenkaasu District Hospital's Blood Bank from January 2010 to December 2018 was conducted.

**Result:**

Cumulatively, HIV and *Treponema pallidum* seropositivity identified in the present study was high (19.1%, [95% C.I (0.026–0.028)]). The prevalence of HIV and syphilis infections were 10.9% (95% C.I (0.098–0.120)) and 8.9% (95% C.I (0.073–0.92)) respectively. Prospective female blood donors were less likely to test positive for *T. pallidum* than males (OR 0.511, [0.340 – 0.769], p=0.001), but the infection was similar among different ages. The data showed downward trend for both HIV and *T. pallidum* seropositivity, (slope=-2.9467, p<0.0001) and (slope=-0.7117, p<0.0001) respectively.

**Conclusion:**

Seroprevalence of HIV and *Treponema pallidum* were high, and their individual or combined seropositivity pose a significant threat to the safety of blood. Extensive and continuous screening for high-risk behaviours and infectious markers before blood donation is therefore Unit, Department of Obstetrics and Gynaecology, College of Medicine, Pan African University of Life and Earth Sciences Institute (PAULESI), University of Ibadan, Ibadan, Nigeria.

## Introduction

Transmission of blood-borne pathogens through blood transfusion is a great threat to mankind and a serious setback in public health, especially in developing countries 1,2. The situation is worsened in sub-Saharan Africa with inadequate infrastructure, insufficiently qualified personnel as well as limited financial resources^3^. In the quest to receive prompt medical intervention through. transfusion, recipients of blood and blood products are at a high risk of acquiring blood-borne pathogens such as human immunodeficiency virus (HIV) and syphilis^1^. The long term likely adverse effects of blood transfusion are likelihoods of endangering the lives of the recipients, their relatives and the entire community at large^4^.

Globally, 37.9 million people were living with HIV at the end of 2018 and about two-thirds of these people live in sub-Saharan Africa^5^. In Africa, previous blood transfusion accounts for between 5–10% of HIV acquisition and this is detrimental to the region (USAIDS, 2010). Several studies have identified high occurrence of HIV among apparently healthy individuals who tend to be blood donors in Ghana and Africa as a continent^1^–^4^,^7^–^11^. The global incidence of syphilis is high at 25.1 cases per 100,000 adult populations. This was estimated from the fifty-five countries that reported in the Global Aids Re sponse Progress Reports (GARPR). Every year, approximately 5.6 million individuals live with syphilis^3^. In Ghana, *Treponema pallidum* has been reported among potential blood donors; 15.3% in Koforidua^12^, 10.42% in Ho 1, and 13.5% in Accra^13^. Owing to the ‘window period’ state of these infectious agents, extensive screening for high-risk behaviours before blood donation must be enforced^14^.

Few studies have been done in Ghana to determine the burden and trend of HIV and syphilis among adult populations, however, no study in this regard has been done in the current study area. Evaluation of the existence of the blood-borne pathogens would help to elucidate the magnitude and trend of the infections in blood donors over the years. Again, the study would provide information on the epidemiology of HIV and syphilis in the study setting.

## Subjects and Methods

A retrospective analysis of secondary data from consecutive prospective voluntary donors, who accessed Nkenkaasu District Hospital's Blood Bank, Ghana from January 2010 to December 2018 was conducted. The study involved male and female participants aged between 17 and 65 years with weight not less than 50kg whose records in the Blood Bank's register were complete. Age, gender, ABO and Rh blood groups and serological results of HIV and syphilis of 3306 healthy donors were reviewed.

### Blood Donor Recruitment

Prospective blood donors were assisted to complete a health questionnaire to either defer or disqualify unsuitable donors while those that satisfied the requirements were screened for blood-borne pathogens. According to the National Blood Transfusion Service's (NBTS) donor health guideline (Form NBTS/DN 20 v2), blood donors are expected to be between 17 and 65 years, mentally competent, physically healthy, and should be devoid of high-risk behaviours, infections, sickle cell, and history of chronic non-communicable diseases. We, therefore, excluded pregnant women, high-risk and test-positive individuals, recipients of recent blood transfusion and those with a history of recent blood-borne infections and other chronic diseases in accordance with the guidelines. Rapid qualitative immunochromatographic test for the detection of antibodies to HIV 1&2 and *Treponema pallidium* in the donors' sera were performed using commercially available kits based on the manufacturers' instructions.

### HIV Assay

Each donor was tested for serum HIV antibodies 1&2 using First Response (HIV 1&2) test kit with 100% sensitivity and specificity (Premier Medical Corporation Private Limited, Kachigam, India). This assay is a lateral flow immunochromatographic technique in which recombinant capture antigens of HIV-1 (gp41 and p24) and HIV-2 (gp36) are incorporated in nitrocellulose membrane, the recombinant antigens are conjugated with colloidal gold particles which react with HIV antibodies present in the serum of blood donors to produce a pink-red colour band. One drop each of donors' serum and assay buffer were added to the sample well of the kit and allowed to run for 15 minutes. A reactive test was confirmed with OraQuick (both sensitivity and specificity being 100%) (OraSure Technologies, Inc., USA).

### Syphilis Assay

**Figure 1:**
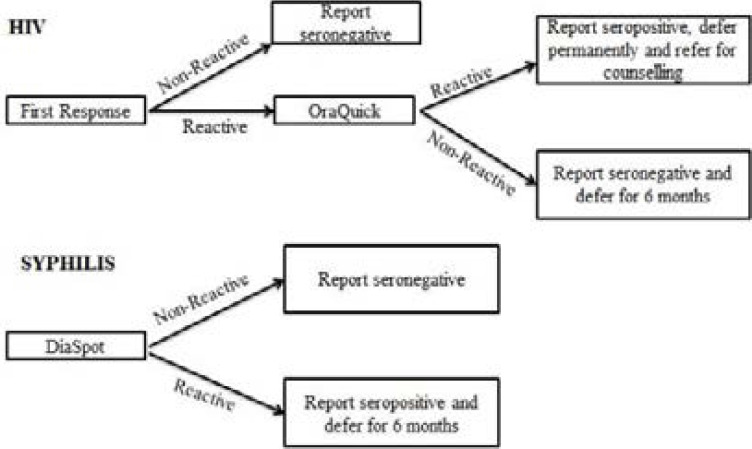
Algorithm for serological screening for HIV and syphilis in Nkenkaasu District Hospital

The sera from the donors were again tested for the presence of Treponema pallidum antibodies using Dias Spot rapid dipstick test strips (DiasSpot Diagnostics, USA). The DiasSpot rapid test kit has relative sensitivity and specificity of 98.6% and 98.5% respectively. The test is a chromatographic immunoassay that uses two purified recombinant antigens of *Treponema pallidum* and gold conjugate in the test band to qualitatively detect anti-TP antibodies. Confirmatory tests were not done after the rapid screening tests indicated the presence of antibodies to *Treponema pallidum*, as this is part of the blood screening protocols before blood donation in resource-limited district hospitals in Ghana. The algorithm used in the blood bank for the screening of blood donors for HIV and syphilis is illustrated in Figure 1.

### Statistical Analysis

Data obtained were analysed with IBM Statistical Package for the Social Sciences (SPSS) version 22.0. Test for normality was done with box plot and Kolmogorov-Smirnoff test for continuous data. Categorical data were descriptively presented as frequencies and proportions, and Chi-square or Fisher's exact tests were used appropriately to determine the differences in proportions. The trend of HIV and syphilis over the nine-years was assessed with the Cochran-Armitage trend test. A p-value < 0.05 was considered statistically significant for all analyses.

## Results

### Demographic and clinical characteristics of the voluntary blood donors

We retrieved 3306 donor records and presented the demographic and blood group characteristics in Table 1. Majority 2739/3306 (82.8%) of the blood donors were males. The minimum age recorded was 17 years and maximum of 65 years, with a mean age of 31.0±8.7 years. A vast proportion (2397/3306, [72.5%]) of the donors were under 36 years, belonged to ABO blood group ‘O’ 2194/3306 (66.4%) and Rh positive (Rh+) 3100/3306 (93.8%).

**Table 1:** Demographic data of voluntary blood donors at Nkenkaasu District Hospital, January 2010–Dec 2018

Variable	Frequency (N)	Percentag
Gender		
Male	2739	82.8
Female	567	17.2
Age		
<36	2397	72.5
36–45	711	21.5
46–55	180	5.4
>55	18	0.5
ABO blood group		
A+/-	408	12.3
B+/-	674	20.4
AB+/-	30	0.9
O+/-	2194	66.4
Rhesus blood group		
Positive	3100	93.8
Negative	206	6.2

### Seropositivity of HIV and *Treponema pallidum* among the donors

The overall prevalence of HIV and syphilis from 2010 to 2018 were 359/3306 (10.9%, [95% CI (0.098–0.120)]) and 271/3306 (8.9%, [95% CI (0.073–0.92)]) respectively. More female blood donors tested positive for HIV 69/567 than males 290/2739 (p=0.271). The number of blood donors that tested positive for HIV in the age category <36 years was 10.7%, 36–45 years was 11.0%, 46–55 years was 11.7% and ≥56 years was 16.7% (p=0.748). Blood donors aged ≥56 years were the highest contributor to HIV positivity 3/18 compared with the other age groups (Table 2). HIV positivity was higher for donors in blood group ‘AB’ (16.7%) compared to blood groups A, B and O of 57/408(14.0%), 65/674(9.6%), 232/2194 (10.6%) respectively (p=0.365). Rh+ donors accounted for 338/359 (94.2%) for HIV positivity and only 21/359 (5.8%) by Rh- donors (p=0.126) (Table 3).

**Table 2:** Distribution of seropositive HIV and Syphilis by Gender and Age blood donors at Nkenkaasu District Hospital from January 2010–Dec 2018

Variables	HIV		Syphilis	

	R N (%)	NR N (%)	R N (%)	NR N (%)
GENDER	p =0.271a		p=0.001a	
Male	290 (10.6)	2449 (89.4)	244 (8.2)	2495 (91.1)
Female	69 (12.2)	512 (90.3)	27 (4.8)	540 (95.2)

Age (years)	p=0.748b		p=0.455b	
<36	257 (10.7)	2140 (89.3)	192 (8.0)	2205 (92.0)
36–45	78 (11.0)	633 (89.0)	67 (9.4)	644 (90.6)
46–55	21 (11.7)	159 (88.3)	11 (6.1)	169 (93.9)

≥55	3 (16.7)	15 (83.3)	1 (5.6)	17 (94.4)
Total	359 (10.9)	2947 (89.1)	271 (8.2)	3306 (91.8)

Data are presented as frequencies with corresponding proportions in parentheses. HIV- Human Immunodeficiency Virus. R-Reactive test, NR-Non-reactive test. n denotes number of positive donors, p denotes p-value, * denotes significant p-value (p<0.05)

**Table 3:** Distribution of seropositive HIV and Syphilis by Gender and Age blood donors at Nkenkaasu District Hospital from January 2010–Dec 2018

Blood type	HCV		Syphilis	

	R N (%)	NR N (%)	R N (%)	NR N (%)
ABO blood group	p =0.365		p=0.477	
A+/-	57 (14.0)	351 (86.0)	39 (9.6)	369 (90.4)
B+/-	65 (9.6)	609 (90.4)	69 (10.2)	605 (89.7)
AB+/-	5 (16.7)	25 (83.3)	1 (3.3)	29 (96.7)
O+/-	232 (10.6)	1962 (89.4)	162 (7.4)	2032 (92.6)

RH blood group	p=0.126		p=0.414	
Positive	338 (10.9)	2762 (89.1)	251 (8.1)	2849 (91.9)
Negative	21 (10.2)	185 (89.8)	20 (9.7)	186 (90.8)

Total	359 (10.9)	2947 (89.1)	271 (8.2)	3306 (91.8)

Data are presented as frequencies with corresponding proportions in parentheses. HIV- Human Immunodeficiency Virus. R-Reactive test, NR-Non-reactive test. n denotes number of positive donors, p denotes p-value, * denotes significant p-value (p<0.05)

The highest prevalence rate of syphilis was seen in male donors 244/2739 (8.2%), whereas female donors had 4.8% prevalence (p=0.001). *T. pallidum* seropositivity was higher in age groups 36–45 and <36 years (9.4% and 8.0% respectively) and least in ≥56-year group 1/18 (5.6%) (p=0.455) (Table 2). With regards to the distribution of the T. pallidum infection within the blood groups, the highest prevalence of 69/674 (10.2%) was observed within donors with blood group B, compared with those having A+/−, O+/− and AB+/− blood types (9.6%, 7.4% and 3.3% respectively) (p=0.271). The Rh-negative group accounted for 9.7% of *T. pallidum* reactive donors) (p=0.414) (Table 3). Females were 48.9% less likely to be Treponema pallidum positive compared with men (OR: 0.511; [95% CI, 0.340–0.769], p=0.001) (Table 4).

**Table 4:** Odd ratios (ORs) and corresponding 95% confidence intervals (95% CIs) for syphilis seropositivity according to Gender among the donors at Nkenkaasu District Hospital from 2010–2018

Characteristics		Syphilis			
				
	N(%)	YES(%)	NO(%)	OR (95% CI)	p
Gender					
Males	2739 (82.9)	244 (8.9)	2495 (91.1)	1.00	<0.001
Females	567 (17.1)	27 (4.8)	540 (95.2)	0.511(0.340, 0.769)	

### Seropositivity of co-infection in the study population

**Figure 2:**
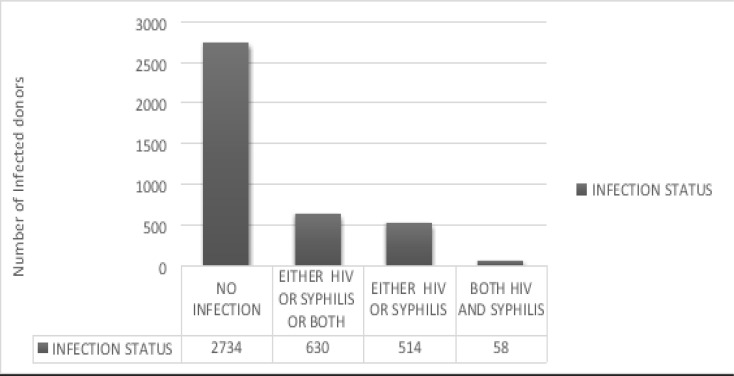
Infection status of voluntary blood donors by HIV and Syphilis at Nkenkaasu District Hospital from 2010–2018

The cumulative seropositivity of HIV and *Treponema pallidum* among the donors was 630/3306 (19.1%, [95% CI (0.026–0.028)]). Of the total donors, 58/3306 (1.75%, [95% CI (0.013–0.023)]) were co-infected (Figure 2).

### Yearly distribution of HIV and Treponema pallidum seropositivity among the voluntary donors over the nine-years

**Figure 3:**
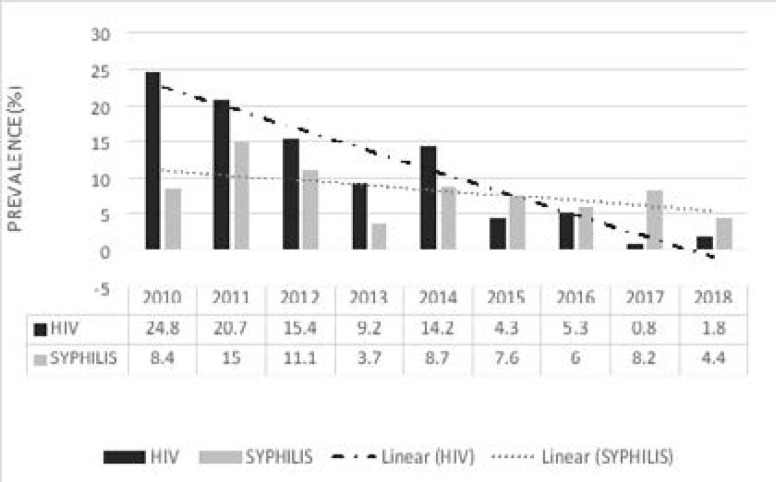
Percentage prevalence of transfusion-transmissible infections in the study period (2010 to 2018) at Nkenkaasu District Hospital

The prevalence of HIV at the hospital decreased from 24.8% in 2010 to 9.2% in 2013 and then increased drastically to 14.2% in 2014. Again, HIV prevalence among the donors showed a decline to 4.3% in 2015, then a mild increase to 5.3% in 2016, followed by a decline to 0.8% in 2017 and finally increased to 1.8% as at 2018 (slope = -2.9467 and p for trends p<0.0001). On the contrary, the *T. pallidum* seropositivity fluctuated yearly throughout the nine-year period. The prevalence increased from 8.4% to 15.0% from 2010 to 2011, accompanied by a slight decline to 11.1% in 2012. We observed a sharp decline from 11.1% to 3.7% in 2013; however, it was followed by a rise in 2014. The prevalence of syphilis decreased from 8.7% in 2004 to 6.0% in 2016 and rose again to 8.2% in 2017; it then decreased to 4.4% in 2018 (slope = -0.7117 and p for trends p<0.0001) (Figure 3).

## Discussion

Blood transfusion poses a high public health risk to recipients of donated blood in sub-Saharan Africa; hence maximum efforts must be made to minimize the risk of transmitting infections to the patients. We, therefore, aimed to determine the prevalence and year-on-year trend of HIV and syphilis infections in voluntary blood donors within Offinso-North district, a rural setting in the Ashanti Region of Ghana. Vast percentage were male donors and below 36 years of age. Most common blood group among donors was O, followed by B while the least common was AB. Rh-positive blood group constituted the majority of the donor blood type as depicted in Table 1. *Treponema pallidum* seropositivity in males was significantly higher than in females (p=0.001); however, its association with age and blood group was not significant. This is contrary to astudy by Bisetegen et al., 11 who recorded rather high *T. pallidum* seroprevalence in females. Also, HIV seropositivity had no association with gender, age and blood type (ABO and Rh). This is similar to a report by Bisetegen et al., 11 but contradicts findings of Mohammadali & Pourfathollah^15^ who reported an association between HIV and ABO group ‘A’ but not with Rh group. The relationship between blood group systems and disease states have been an issue of speculation, as actual mechanisms are not well elucidated. However, it has been explained that ABO blood group system is made of carbohydrate antigens^16^, and some viruses, as well as bacteria, mask themselves with carbohydrate substances to hide their antigenic regions and prevent destruction by the specific adaptive immune mechanisms; ABO antigens have since been found on glycoprotein 120, a surface protein present on the envelope of HIV type I^17^.

Cumulatively, HIV and *T. pallidum* seropositivity identified in the present study was high (19.1%, [95% CI (0.026–0.028)]). This seroprevalence is similar to 19.3% obtained from an earlier study conducted in Kano, Nigeria^18^, but relatively lower than 21.2% reported in first-time donors in Edea, Cameroon^7^. Conversely, the seropositivity of HIV and *T. pallidum* in our study was higher than reports from other sub-Saharan African countries: 9.4% in Western Kenya^19^, and 6.55% in Northwest Ethiopia^20^. The high TTI seroprevalence in our study could be due to different periods within which the studies were conducted and/or different sensitivities of test kits employed to screen the donors. Whereas we studied blood donors over nine-year period, some of the comparing studies were conducted over short periods: one^19^ and three^20^ years respectively.

Our findings showed a high (10.9%, [95% CI (0.098–0.120)] seroprevalence of HIV infection, which is similar to 11.7% reported in Northwest Ethiopia^21^, but higher than findings obtained from similar studies within the same country, Ghana: in Ho (4.78%)^22^, Akwatia (4.60%)^1^ and Kintampo (5.0%)^9^. This variation in prevalence within the same country could have resulted from the different settings within which the studies were conducted and the screening methods. Whereas the Ho, Akwatia, Kintampo and Accra studies were conducted in urban locations, our study was in a rural district. Most of the urban hospitals utilize HIV-p24 antigen testing, which is known to greatly reduce the residual risk of HIV infection from 0.38 to 0.24 per million^23^,^24^. This is not the case in most rural areas like this facility that continues to depend on antibody screening methods. Therefore, as long as these rural facilities continue to transfuse anti-HIV negative blood which may be HIV infected as a result of antibody screening during the “window period phase”, the residual risk of HIV may continue to rise in our environment. The high HIV seropositivity in this current study contrasts with findings from similar studies conducted in urban locations and developed economies in other parts of Africa: Calabar, Nigeria (4.2%)^3^, Northwest Ethiopia (2.24%)^20^, Douala, Cameroon (1.8%)^25^, Edéa, Cameroon (4.1%)^7^, Southwest Nigeria (3.1%)^26^, and 3.8% in both Ethiopia^27^ and Dar es Salaam, Tanzania^28^. Whereas this current study involved entirely voluntary donors, most of the studies conducted in other African countries also included family replacement and/or commercial donors: the studies from Northwest Ethiopia^20^ and Tanzania^28^ recruited predominantly family replacement donors (94.1% and 70.4% respectively), and the Cameroon study, first-time donors 7. Researches conducted in several countries have proven that blood collected from the volunteer, non-remunerated donors is much safer than blood collected from replacement donors^29^. HIV and syphilis infections are most likely to be recorded in blood from replacement donors because relatives are most likely to deny certain high-risk lifestyles^30^. This could explain why a similar study in South Ethiopia which recruited entirely voluntary donors reported slightly increased HIV seroprevalence of 6.4%^11^, although not as high as the 10.9% recorded in our study. The high HIV seroprevalence in this current study could also be attributed to variations within populations, in the sense that our study was based on prevalence from a single district hospital located in a rural setting where people have similar population characteristics and exposures, while most of the contrasting reports were conducted in referral healthcare facilities 7,19,25–28 where people from diverse backgrounds seek healthcare.

Individually, the overall seropositivity of antibody to *T. pallidum* accounted for 8.9% [95% CI (0.073–0.92)] of infections in the present study. This corroborates 8.1% found in Douala, Cameroon^25^; 7.9% in South Ethiopia 11; and 7.5% in Kano, Nigeria (18). However, in Eastern Region of Ghana, a slightly increased syphilis prevalence of 10.42% was recorded^1^; again a higher rate (13.5%) has been found in the country's capital city 13. In the same country, Ghana, 2.46% has recently been reported 22. The screening test for syphilis is the same across district blood centre laboratories in Ghana but the plausible explanation to the variation in the prevalence rate of syphilis in Ghana could be differences in sexual behaviours and increased ratio of family replacement to voluntary donors. Syphilis and HIV are transmitted through sexual contacts^31^, and because of this commonality in the transmission of both diseases, high seropositivity in one could, therefore, influence the occurrence of the other^11^. This could justify why the seropositivity of both infections was marked in the present study. The discordance with other Ghanaian studies, however, may be because the study by Ampofo et al.,^13^ recruited predominantly (96.3%) family replacement donors, with only 3.7% voluntary donors. The Akwatia study, on the other hand, compared sentinel data of pregnant women and blood donors^22^. Conversely, in other parts of Africa, much reduced prevalence have been reported: 4.7% (28), 1.1%^26^, and 3.98%^32^. Treponema pallidum seropositivity at Nkenkaasu hospital was extremely higher compared to reports in parts of Asia^33^,^34^ which could be attributed to varying exposure rates to risk factors. In the present study, prospective female blood donors were significantly less likely to test positive for *T. pallidum* than males. *Treponema pallidum* seropositivity in 36–45-year bracket was the highest (9.4%), however, the difference among different ages groups was not significant. Moreover, the likelihood to be infected with HIV did not vary significantly with age and gender.

The nine years under review (2010–2018) showed a downward trend for HIV and Treponema pallidum seropositivity. The trend test for HIV infection prevalence resulted in -2.947 regression slope (p for trends p<0.0001). *Treponema pallidum* seropositivity also showed a significant decline according to the test with a regression slope of -0.7117 (p for trends p<0.0001). This decline over the period may be the result of increasing awareness of diseases transmissible via transfusion, improvement in pre-donation screening^34^, campaigns which seek to sensitize the public on the need to voluntarily donate blood, and improved deferral system in which a structured questionnaire is administered to exclude high-risk donors across Ghana in recent years. The reducing trend is consistent with findings from a similar study in Africa^27^. Contrary to our finding, syphilis trend was found to significantly increase over 4 years in the same country, Ghana^1^. The spread of syphilis according to Zhu et al.,^35^ is influenced by biological and social factors which may vary across populations in different geographical locations. Syphilis co-infection with HIV was seen in 1.75% of the donors. However, in a similar study conducted in Northwest Ethiopia, HIV-Syphilis co-infection was the combination with the highest frequency (38%) among blood donors that had more than one infection^27^. The algorithms used to detect HIV and Treponema pallidum in this study have been used in a similar study^3^ and found to be appropriate especially in resource-limited settings like the study area.

This study was not without limitations: only voluntary blood donors whose complete details were found in the donor register of the Blood Bank were sampled for the study and this may have altered the overall and yearly burden of the infectious markers among blood donors in this setting. Again, the retrospective nature of the study did not afford to differentiate first-time and repeat donors; a study in China by Ji et al.,^34^ suggests that blood-borne infectious markers are more common in first-time blood donors making them a high-risk group. Differentiating between the two groups would have helped to identify which group poses the greatest risk to blood safety and contributor to the overall burden of the infections. Also, there was no confirmatory test for *Treponema pallidum* in the study setting, the high prevalence, therefore, could have been exaggerated as we relied only on a rapid screening test that has the potential to detect other closely related Treponema species.

## Conclusion

Collectively, this study identified high (19.1%) HIV and Treponema pallidum seropositivity. Also, the individual seroprevalence of HIV and syphilis was high and showed downward trends over the nine years. These infectious markers, either combined or individually pose a significant threat to blood safety. Extensive screening for highrisk behaviours before blood donation is therefore imperative and must be enforced, especially in rural settings where resources are limited to eradicate or at least lessen the danger of transmitting the infections. We recommend continuous and complete adherence to standard operating procedures for blood donor selection. We implore authorities of health agencies in Ghana to resource blood centre laboratories with equipment to perform HIV nucleic acid testing as being practised elsewhere.
